# Lifestyle factors and hyperuricemia risk: a prospective cohort study of 14,635 participants examining the protective role of daily stair climbing

**DOI:** 10.3389/fnut.2025.1635746

**Published:** 2025-08-06

**Authors:** Wenkui Yin, Donglin Luo, Wenying Huang, Haichao Jiang, Yahai Wang, Haixia Qi

**Affiliations:** ^1^College of Physical Education, Wuha City Polytechnnic, Wuhan, China; ^2^College of Arts and Physical Education, Nanchang Normal College of Applied Technology, Nanchang, China; ^3^Faculty of Health Service, Naval Medical University, Shanghai, China; ^4^College of Physical Education, Yangzhou University, Yangzhou, China; ^5^College of Physical Education, Jiangxi Normal University, Nanchang, China

**Keywords:** stair climbing, uric acid, blood lipid biomarkers, prospective cohort study, the UK biobank

## Abstract

**Background:**

This study aimed to explore the association between daily stair climbing and the risk of hyperuricemia and to investigate the potential mediating role of blood lipid biomarker levels in this association.

**Methods:**

This study is a prospective cohort study from the UK Biobank, including 14,635 participants. Participants were categorized based on their self-reported daily stair climbing habits and the presence of hyperuricemia, which was defined as serum uric acid concentration > 420 μmol/L for men and > 360 μmol/L for women. Blood lipid biomarker levels were assessed as potential mediators. We used a mediation analysis framework to estimate the direct and indirect effects of daily stair climbing on hyperuricemia risk. All analyses were conducted using R Studio version 4.2.3. Statistical significance was defined as a two-sided *p*-value of < 0.05.

**Results:**

Overall, compared to the no stair climbing group, with full adjustment, we observed a significant negative correlation between participants who climbed 160 to 200 steps of stairs daily and hyperuricemia; the HRs were 0.70 (95% CI: 0.51–0.95, *p* = 0.024). Mediation analysis revealed a significant indirect effect of stair climbing (160–200 steps/day) on the risk of hyperuricemia, mediated through high-density lipoprotein cholesterol and triglyceride levels, with mediation proportions of 27.6 and 21.8%, respectively (*p* < 0.001).

**Conclusion:**

Daily stair climbing is associated with a reduced risk of hyperuricemia, and this relationship may be partially mediated by alterations in high-density lipoprotein cholesterol and triglyceride levels. These findings suggest that promoting daily physical activity, such as stair climbing, may be an effective strategy for managing uric acid levels and reducing the risk of hyperuricemia.

## Introduction

Hyperuricemia—characterized by elevated serum uric acid (SUA)—is an emerging metabolic risk factor for gout, cardiovascular diseases, and chronic kidney disease (1, 2). Its pathogenesis involves disrupted uric acid metabolism, modulated by diet habits (3), renal function (4), genetic predisposition (5, 6), and lifestyle factors such as physical activity (7–10). Among these, physical inactivity is a modifiable risk factor that has been suggested to contribute to the pathophysiology of hyperuricemia. Regular physical activity is known to benefit cardiovascular health, improve metabolic function, and enhance renal health, potentially mitigating the risk of developing hyperuricemia (11). Stair climbing is a common, low-cost, and easily accessible form of exercise that engages multiple muscle groups, improves cardiovascular function, and can be incorporated into daily routines without the need for specialized equipment (12–15). While its cardiovascular benefits are established (16–18), stair climbing’s role in SUA levels and its potential role in preventing or managing hyperuricemia remain to be fully understood. Thus, investigating the relationship between stair climbing and hyperuricemia could provide valuable insights into the role of simple, everyday physical activities in metabolic health.

Several studies have explored the broader relationship between physical activity and hyperuricemia. It was reported that higher levels of physical activity were inversely associated with serum uric acid concentrations (7), suggesting that more active individuals may have a lower risk of developing hyperuricemia. Stair climbing, being a more routine and less deliberate activity, may serve as a proxy for general physical activity in the daily lives of individuals and could provide a unique insight into how everyday movements influence metabolic outcomes. Moreover, a growing body of evidence suggests that physical activities, even those that occur in short bursts throughout the day, can have significant impacts on hyperuricemia or metabolic health (7, 19–21). Stair climbing, as an activity that can be performed in short intervals and incorporated seamlessly into daily routines, may be a particularly effective form of exercise for improving metabolic health outcomes in the general population.

This study aims to investigate the potential association between daily stair climbing and hyperuricemia risk in the UK Biobank cohort. We also aim to explore whether the potential benefits of stair climbing are more pronounced in certain subgroups of the population, such as those with unhealthy diets or sedentary lifestyles. By leveraging the extensive data from the UK Biobank, we provided a deeper understanding of how a simple, daily activity, such as stair climbing, can contribute to the prevention of hyperuricemia, potentially informing public health strategies aimed at reducing the burden of this condition.

## Methods

### Data source and study population

The UK Biobank is a population-based prospective cohort comprising over 500,000 participants aged 37–73 years recruited across the United Kingdom between 2006 and 2010. Detailed techniques for this research are available in a previous study (22). The study protocol was approved by the National Health Service (NHS) National Research Ethics Service (Ref: 11/NW/0382), with all participants providing written informed consent. Detailed information about the UK Biobank cohort is available on their website.^1^ Our study was conducted under UK Biobank application number 75732.

**Figure 1:**
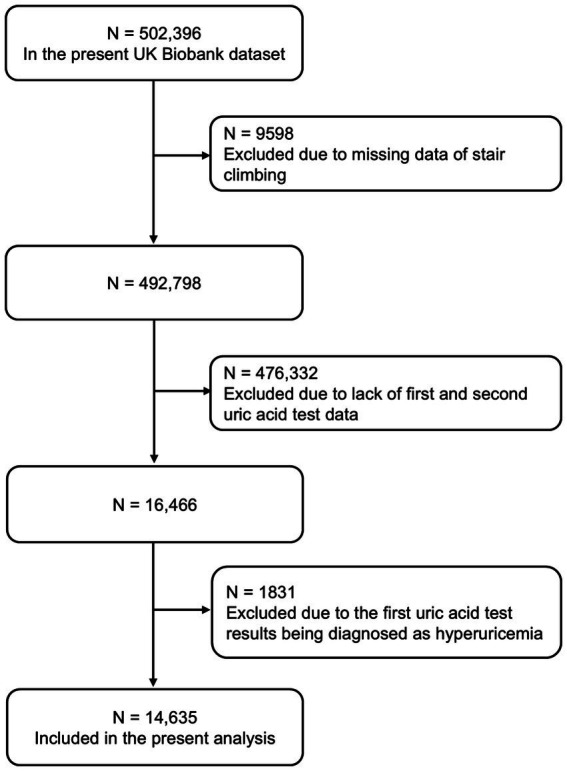
Flowchart of participants’ selection.

We excluded individuals with missing data on stair climbing (*n* = 9,598), with unavailable uric acid measurements in phase I and phase II (*n* = 476,332), and subjects with pre-existing hyperuricemia at baseline (*n* = 1831). Finally, we included 14,635 participants in the present study (Figure 1).

### Assessment of hyperuricemia diseases

Hyperuricemia is a metabolic disease caused by an increase in uric acid levels in the blood, due to the metabolic disorder of a substance called purine in the human body. The reference value of serum uric acid concentration was > 420 for men and > 360 for women (23). The end of follow-up was defined as the earliest occurrence of hyperuricemia until 03 October 2017, whichever occurred first.

### Assessment of stair climbing

Stair climbing exposure was assessed through self-reported frequency using the following standardized question: “At home, during the last 4 weeks, about how many times a DAY do you climb a flight of stairs?” (approximately 10 steps). The daily number of steps climbed (steps/day) was determined by multiplying the frequency of climbing one flight of stairs per day by 10 steps. Then, daily stair climbing was classified into six groups: none, 10–50, 60–100, 110–150, 160–200, and over 200 steps/day. Participants with missing stair climbing data were excluded from all analyses.

### Other covariates

We considered sociodemographic factors (age, sex, ethnicity, Townsend deprivation index, education qualification, and employment), lifestyle factors (smoking status, alcohol drinker status, sleep duration, sedentary time, vegetable and fruit intake, and total physical activity), and health conditions [body mass index (BMI)] as potential confounders. The vegetable and fruit intake was converted into proportions according to the guidelines on the NHS website (24). The definition and category of covariates are presented in Table 1.

**Table 1:** Characteristics of hyperuricemia participants grouped by daily stair climbing.

Characteristics, number (%) /mean (SD)	Stair climbing (steps/day)						
	None (*N* = 977)	10–50 (*N* = 2,385)	60–100 (*N* = 5,609)	110–150 (*N* = 3,117)	160–200 (*N* = 1,409)	> 200 (*N* = 1,138)	Overall (*N* = 14,635)
Follow-up years	8.27 [5.57, 10.4]	8.34 [5.52, 10.4]	8.36 [5.48, 10.4]	8.34 [5.55, 10.4]	8.38 [5.55, 10.4]	8.33 [5.61, 10.3]	8.35 [5.48, 10.4]
Hyperuricemia incident	77 (7.9%)	196 (8.2%)	431 (7.7%)	232 (7.4%)	84 (6.0%)	91 (8.0%)	1,111 (7.6%)
Age, years	59.8 (6.64)	56.6 (7.42)	56.9 (7.35)	57.1 (7.53)	57.0 (7.47)	56.8 (7.56)	57.1 (7.42)
Female	490 (50.2%)	1,176 (49.3%)	2,846 (50.7%)	1,663 (53.4%)	767 (54.4%)	636 (55.9%)	7,578 (51.8%)
Male	487 (49.8%)	1,209 (50.7%)	2,763 (49.3%)	1,454 (46.6%)	642 (45.6%)	502 (44.1%)	7,057 (48.2%)
White ethnicity	960 (98.3%)	2,310 (96.9%)	5,476 (97.6%)	3,059 (98.1%)	1,374 (97.5%)	1,112 (97.7%)	14,291 (97.6%)
Townsend deprivation index	−2.13 (2.91)	−1.63 (2.96)	−2.13 (2.55)	−2.21 (2.52)	−2.24 (2.57)	−2.10 (2.77)	−2.07 (2.67)
College or above degree	305 (31.2%)	918 (38.5%)	2,478 (44.2%)	1,491 (47.8%)	707 (50.2%)	509 (44.7%)	6,408 (43.8%)
Employed	414 (42.4%)	1,547 (64.9%)	3,519 (62.7%)	1761 (56.5%)	769 (54.6%)	626 (55.0%)	8,636 (59.0%)
Never smoking	527 (53.9%)	1,366 (57.3%)	3,388 (60.4%)	1866 (59.9%)	885 (62.8%)	747 (65.6%)	8,779 (60.0%)
Alcohol consumption	914 (93.6%)	2,220 (93.1%)	5,325 (94.9%)	2,934 (94.1%)	1,339 (95.0%)	1,065 (93.6%)	13,797 (94.3%)
Vegetables and fruit (≥ 5/week)	383 (39.2%)	821 (34.4%)	2,125 (37.9%)	1,350 (43.3%)	632 (44.9%)	557 (48.9%)	5,868 (40.1%)
Sedentary behavior, hours	4.58 (2.49)	4.47 (2.57)	4.30 (2.36)	4.18 (2.34)	4.06 (2.30)	4.07 (2.60)	4.28 (2.42)
Sleep duration, hours	7.22 (1.34)	7.15 (1.13)	7.15 (1.11)	7.19 (1.03)	7.19 (1.17)	7.13 (1.10)	7.17 (1.12)
Physical activity (MET-min/week)	2,590 (2580)	2,290 (2530)	2,350 (2370)	2,490 (2410)	2,790 (2500)	3,510 (2970)	2,520 (2500)
BMI, kg/m^2^	27.0 (4.46)	27.4 (4.77)	26.6 (4.23)	26.1 (4.00)	25.9 (3.90)	25.5 (3.71)	26.5 (4.26)

BMI, body mass index; IQR, interquartile range.

### Statistical analysis

Baseline characteristics were stratified by daily stair climbing categories and presented as counts (percentages) for categorical variables or mean ± standard deviation (SD) for continuous variables. We used Cox proportional hazards regression models to predict the association between daily stair climbing and incident hyperuricemia. Hazard ratios (HRs) and corresponding 95% confidence intervals (95% CIs) were calculated, and the proportional hazards assumption was validated using Schoenfeld residuals. Person years were calculated from baseline until the end of follow-up. Three incremental models were constructed using the no stair climbing group as reference: Model 1 was adjusted for age and sex; Model 2 was adjusted for Model 1 plus sociodemographic factors (ethnicity, socioeconomic status, education, and employment status); and Model 3 was adjusted for Model 2 plus lifestyle factors (cigarette smoking, vegetable and fruit intake, alcohol consumption, body mass index, sedentary behavior, sleep duration, and total physical activity).

To quantify the indirect (mediated) effect of blood lipid biomarkers, we followed the guidelines by Preacher and Hayes (25) and utilized the mediation package in R to compute the indirect effect. The total effect, direct effect, and indirect effect were estimated as follows: total effect (TE): The total association between stair climbing (X) and hyperuricemia risk (Y), without considering the mediator; direct effect (DE): The effect of stair climbing (X) on hyperuricemia risk (Y), controlling for blood lipid biomarker levels; and indirect effect (IE): The portion of the effect of stair climbing (X) on hyperuricemia risk (Y) that is mediated through blood lipid biomarker levels (M). The mediation proportion was calculated as IE/TE × 100%. Sensitivity analyses by removing participants with missing values of covariates were conducted to test the robustness of our main findings. R Studio version 4.2.3 was used for analyses. Statistical significance was defined as a two-sided *p*-value of < 0.05.

## Results

### Population characteristic

Table 1 presents the baseline characteristics of participants stratified by daily stair climbing levels. Our analysis included 14,635 participants (mean age 57.1 years; 51.8% female). During a mean follow-up period of 8.35 years, a total of 1,111 incident hyperuricemia cases were documented. Participants with college degrees or higher education were most prevalent in the 160–200 steps/day group. Currently employed individuals showed higher stair climbing frequency. Over 90% of participants reported alcohol consumption. Vegetable and fruit intake (≥ 5 servings/week) increased with daily stair climbing volume. Daily stair climbing was inversely associated with sedentary time, sleep duration, and BMI but positively associated with overall physical activity levels.

### Associations between daily stair climbing and risk of hyperuricemia disease

The independent associations between daily stair climbing and incident hyperuricemia are presented in Table 2. After full adjustment for potential confounders, participants who climbed 160–200 steps/day demonstrated a significantly lower risk of hyperuricemia compared to non-climbers (HRs = 0.70, 95% CI: 0.51–0.95, *p* = 0.024).

**Table 2:** Association between daily stair climbing and risk of hyperuricemia disease in 14,635 participants.

Stair climbing (steps/day)	Hazard ratio (95% CI), *p*-value					
	Model 1		Model 2		Model 3	
None	1.00 (reference)		1.00 (reference)		1.00 (reference)	
10–50	0.90 (0.69, 1.18)	0.241	0.92 (0.71, 1.2)	0.554	0.88 (0.68, 1.15)	0.366
60–100	0.89 (0.69, 1.13)	0.327	0.90 (0.71, 1.15)	0.406	0.92 (0.72, 1.17)	0.493
110–150	0.86 (0.67, 1.12)	0.262	0.87 (0.67, 1.13)	0.308	0.92 (0.71, 1.20)	0.546
160–200	**0.64 (0.47, 0.87)**	**0.005**	**0.66 (0.48, 0.90)**	**0.008**	**0.70 (0.51, 0.95)**	**0.024**
> 200	0.97 (0.72, 1.32)	0.861	0.99 (0.73, 1.35)	0.963	1.15 (0.84, 1.56)	0.381

The values in bold denotes significant differences (*p* < 0.05). CI, Confidence interval. Model 1 was adjusted for age and sex; Model 2 was adjusted for age, sex, ethnicity, socioeconomic status, education, and employment status; Model 3 was adjusted for Model 2 plus cigarette smoking, vegetable and fruit intake, alcohol consumption, body mass index, sedentary behavior, sleep duration, and total physical activity.

### Secondary analysis

The results of the subgroup analyses are shown in Table 3. Significant inverse associations between daily stair climbing (160–200 steps/day) and hyperuricemia risk were observed only in participants aged 45–65 years (HRs = 0.67, 95% CI: 0.47–0.96, *p* = 0.028) and those with normal BMI (HRs = 0.42, 95% CI: 0.21–0.85, *p* = 0.015). Specifically, women demonstrated reduced hyperuricemia risk at 60–100 steps/day (HRs = 0.64, 95% CI: 0.43–0.94, *p* = 0.022), whereas men showed a benefit at 160–200 steps/day (HRs = 0.63, 95% CI: 0.41–0.97, *p* = 0.035). Notably, among all subgroup analyses, only BMI demonstrated a significant interaction effect on the inverse association between stair climbing and hyperuricemia risk (*p* for interaction = 0.034). However, these subgroup findings should be interpreted with caution due to the wide confidence intervals reflecting limited statistical power due to low hyperuricemia incidence (7.6%).

**Table 3:** Subgroup analyses of associations between daily stair climbing and risk of hyperuricemia disease.

Stair climbing (steps/day)	Hazard ratio (95% CI), *p*-value					
	Model 1		Model 2		Model 3	
Age						
Age <= 45 years (*N* = 1,413)						
None	1.00 (reference)		1.00 (reference)		1.00 (reference)	
10–50	1.61 (0.37, 7.06)	0.526	1.56 (0.36, 6.89)	0.554	0.98 (0.22, 4.41)	0.975
60–100	1.81 (0.44, 7.53)	0.413	1.63 (0.39, 6.82)	0.501	1.19 (0.28, 5.05)	0.812
110–150	1.77 (0.42, 7.53)	0.437	1.54 (0.36, 6.58)	0.560	1.07 (0.24, 4.69)	0.926
160–200	1.62 (0.34, 7.63)	0.544	1.42 (0.3, 6.74)	0.657	1.02 (0.21, 4.97)	0.976
> 200	1.86 (0.4, 8.63)	0.427	1.69 (0.36, 7.86)	0.503	1.58 (0.33, 7.55)	0.568
45 < Age < 65 (*N* = 11,424)						
None	1.00 (reference)		1.00 (reference)		1.00 (reference)	
10–50	0.87 (0.65, 1.18)	0.372	0.9 (0.67, 1.22)	0.506	0.87 (0.65, 1.18)	0.380
60–100	0.85 (0.65, 1.12)	0.250	0.88 (0.67, 1.16)	0.361	0.92 (0.70, 1.22)	0.570
110–150	0.79 (0.59, 1.06)	0.113	0.81 (0.60, 1.08)	0.153	0.88 (0.66, 1.19)	0.416
160–200	**0.59 (0.41, 0.84)**	**0.003**	**0.61 (0.43, 0.87)**	**0.006**	**0.67 (0.47, 0.96)**	**0.028**
> 200	0.86 (0.61, 1.22)	0.403	0.89 (0.62, 1.26)	0.507	1.06 (0.74, 1.51)	0.748
Age > = 65 (*N* = 1798)						
None	1.00 (reference)		1.00 (reference)		1.00 (reference)	
10–50	0.90 (0.47, 1.73)	0.744	0.86 (0.44, 1.67)	0.653	0.86 (0.44, 1.68)	0.655
60–100	0.86 (0.48, 1.54)	0.616	0.81 (0.45, 1.46)	0.492	0.76 (0.42, 1.38)	0.364
110–150	1.12 (0.61, 2.04)	0.723	1.11 (0.60, 2.05)	0.730	1.12 (0.60, 2.08)	0.715
160–200	0.76 (0.35, 1.62)	0.469	0.71 (0.33, 1.54)	0.386	0.74 (0.34, 1.61)	0.446
> 200	1.54 (0.77, 3.09)	0.221	1.44 (0.71, 2.91)	0.307	1.63 (0.80, 3.36)	0.181
*p* for interaction (age*stair climbing)	0.388		0.374		0.439	
Sex						
Female (*N* = 7,578)						
None	1.00 (reference)		1.00 (reference)		1.00 (reference)	
10–50	0.76 (0.50, 1.15)	0.187	0.79 (0.52, 1.20)	0.275	0.69 (0.46, 1.05)	0.087
60–100	**0.61 (0.42, 0.89)**	**0.010**	**0.63 (0.43, 0.93)**	**0.019**	**0.64 (0.43, 0.94)**	**0.022**
110–150	0.71 (0.47, 1.05)	0.087	0.74 (0.50, 1.11)	0.142	0.81 (0.54, 1.20)	0.291
160–200	0.72 (0.46, 1.14)	0.159	0.76 (0.48, 1.21)	0.245	0.88 (0.55, 1.40)	0.584
> 200	**0.59 (0.36, 0.98)**	**0.039**	0.62 (0.38, 1.03)	0.066	0.82 (0.49, 1.36)	0.430
Male (*N* = 7,057)						
None	1.00 (reference)		1.00 (reference)		1.00 (reference)	
10–50	1.05 (0.74, 1.48)	0.800	1.06 (0.75, 1.50)	0.749	1.06 (0.75, 1.50)	0.742
60–100	1.12 (0.82, 1.55)	0.478	1.13 (0.82, 1.56)	0.449	1.16 (0.84, 1.61)	0.360
110–150	0.99 (0.70, 1.40)	0.962	0.99 (0.70, 1.39)	0.948	1.03 (0.73, 1.46)	0.858
160–200	**0.60 (0.39, 0.92)**	**0.018**	**0.61 (0.40, 0.93)**	**0.021**	**0.63 (0.41, 0.97)**	**0.035**
> 200	1.33 (0.90, 1.96)	0.153	1.35 (0.91, 1.99)	0.134	1.52 (1.02, 2.25)	0.037
*p* for interaction (sex*stair climbing)	0.446		0.480		0.884	
BMI						
Normal (*N* = 5,839)						
None	1.00 (reference)		1.00 (reference)		1.00 (reference)	
10–50	0.60 (0.34, 1.05)	0.075	0.62 (0.35, 1.11)	0.105	0.66 (0.37, 1.18)	0.164
60–100	0.64 (0.39, 1.05)	0.076	0.65 (0.39, 1.07)	0.092	0.73 (0.44, 1.21)	0.221
110–150	**0.51 (0.30, 0.88)**	**0.014**	**0.53 (0.31, 0.91)**	**0.022**	0.59 (0.34, 1.02)	0.057
160–200	**0.34 (0.17, 0.67)**	**0.001**	**0.36 (0.18, 0.73)**	**0.004**	**0.42 (0.21, 0.85)**	**0.015**
> 200	0.62 (0.33, 1.17)	0.143	0.67 (0.35, 1.26)	0.213	0.80 (0.42, 1.53)	0.503
Overweight or obese (*N* = 8,705)						
None	1.00 (reference)		1.00 (reference)		1.00 (reference)	
10–50	0.98 (0.72, 1.32)	0.871	0.99 (0.73, 1.34)	0.954	0.96 (0.71, 1.3)	0.784
60–100	0.99 (0.75, 1.31)	0.956	1.00 (0.76, 1.33)	0.973	0.99 (0.75, 1.31)	0.933
110–150	1.04 (0.78, 1.40)	0.784	1.03 (0.77, 1.39)	0.829	1.04 (0.77, 1.40)	0.818
160–200	0.80 (0.57, 1.14)	0.223	0.81 (0.57, 1.15)	0.240	0.81 (0.57, 1.16)	0.252
> 200	1.24 (0.88, 1.76)	0.218	1.25 (0.88, 1.77)	0.209	1.31 (0.92, 1.86)	0.131
*p* for interaction (BMI*stair climbing)	0.016		0.030		0.034	
Alcohol						
Current (*N* = 13,797)						
None	1.00 (reference)		1.00 (reference)		1.00 (reference)	
10–50	0.89 (0.68, 1.17)	0.484	0.91 (0.69, 1.19)	0.484	0.87 (0.66, 1.14)	0.298
60–100	0.88 (0.69, 1.13)	0.382	0.89 (0.7, 1.15)	0.382	0.90 (0.7, 1.16)	0.420
110–150	0.85 (0.65, 1.11)	0.270	0.86 (0.66, 1.12)	0.270	0.90 (0.69, 1.18)	0.444
160–200	**0.65 (0.48, 0.89)**	**0.013**	**0.67 (0.49, 0.92)**	**0.013**	**0.71 (0.51, 0.97)**	**0.030**
> 200	0.99 (0.72, 1.34)	0.973	1.01 (0.74, 1.37)	0.973	1.15 (0.84, 1.57)	0.375
Never (*N* = 450)						
None	1.00 (reference)		1.00 (reference)		1.00 (reference)	
10–50	1.58 (0.34, 7.24)	0.557	2.19 (0.44, 10.94)	0.339	1.28 (0.23, 7.23)	0.783
60–100	0.46 (0.09, 2.3)	0.343	0.56 (0.10, 3.03)	0.504	0.60 (0.1, 3.76)	0.588
110–150	0.70 (0.13, 3.63)	0.670	0.74 (0.14, 3.92)	0.721	0.57 (0.09, 3.72)	0.556
160–200	0.37 (0.03, 4.19)	0.423	0.52 (0.04, 6.14)	0.604	1.16 (0.08, 16.26)	0.911
> 200	0.52 (0.07, 3.71)	0.511	0.63 (0.08, 4.86)	0.655	0.90 (0.10, 8.2)	0.928
Previous (*N* = 383)						
None	1.00 (reference)		1.00 (reference)		1.00 (reference)	
10–50	0.34 (0.02,5.63)	0.450	0.27 (0.02,4.74)	0.373	0.05 (0, 1.97)	0.110
60–100	2.59 (0.32, 20.75)	0.370	3.01 (0.34,26.61)	0.321	3.68 (0.34, 39.57)	0.283
110–150	1.83 (0.21, 16.32)	0.587	1.69 (0.18,16.09)	0.649	2.50 (0.21, 30.22)	0.471
160–200	0.00 (0.00, Inf)	0.998	0.00 (0.00, Inf)	0.998	0.00 (0.00, Inf)	0.998
> 200	1.28 (0.07, 21.85)	0.865	1.58 (0.09, 28.88)	0.759	0.09 (0.00, 7.54)	0.290
*p* for interaction (alcohol*stair climbing)	0.664		0.683		0.693	

The value in bold denotes significant differences (*p* < 0.05). CI, Confidence interval. Model 1 was adjusted for age and sex; Model 2 was adjusted for age, sex, ethnicity, socioeconomic status, education, and employment status; Model 3 was adjusted for Model 2 plus cigarette smoking, vegetable and fruit intake, alcohol consumption, body mass index, sedentary behavior, sleep duration, and total physical activity.

### Mediation analysis

**Figure 2:**
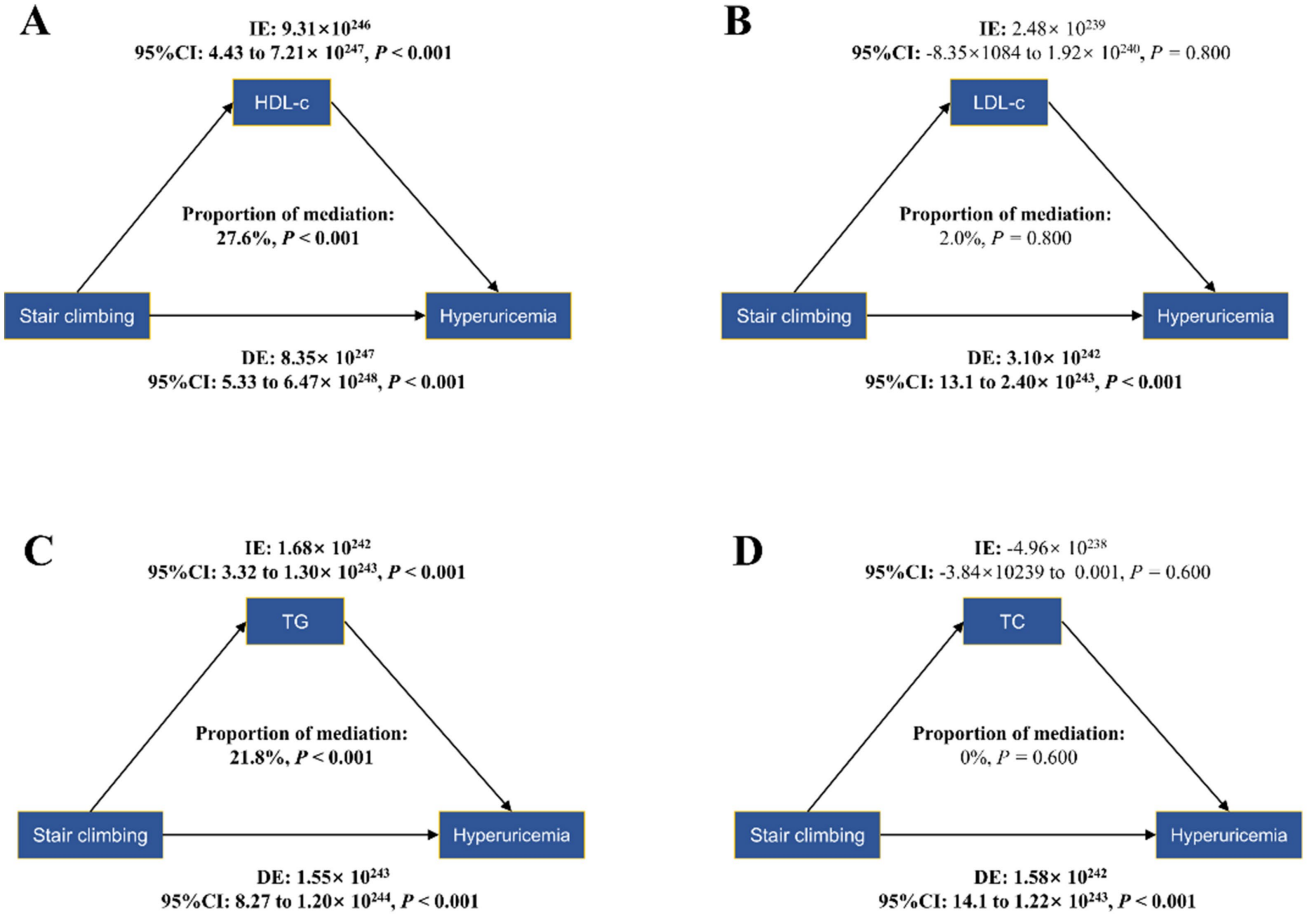
Mediation effects of lipid profiles in the stair-climbing-hyperuricemia association: high-density lipoprotein cholesterol **(A)**, low-density lipoprotein cholesterol **(B)**, triglyceride **(C)**, and total cholesterol **(D)**. The figure presents bootstrap mediation analyses examining potential mediating effects of serum lipids on the association between daily stair climbing (160–200 steps/day) and hyperuricemia incidence. Models were adjusted for age and sex, ethnicity, socioeconomic status, education, and employment status, cigarette smoking, vegetable and fruit intake, alcohol consumption, body mass index, sedentary behavior, sleep duration, and total physical activity. CI, confidence interval; indirect effect (IE), estimated effect through the mediator; direct effect (DE), the estimate of the direct effect; proportion of mediation = IE/DE + IE, ratio expressed as percentage.

To further explore the association between daily stair climbing and the risk of hyperuricemia, a mediation analysis was conducted to explore the mediating effect of blood lipid biomarkers. The mediating role of blood lipid biomarkers in the association between daily stair climbing and hyperuricemia risk is shown in Figure 2 and Table 4. The biomarkers of high-density lipoprotein cholesterol and triglyceride significantly mediated the association between daily stair climbing and hyperuricemia risk, with a proportion of mediation of 27.6 and 21.8%, respectively (*p* < 0.001).

**Table 4:** Mediation analysis of lipid biomarkers between stair climbing (160–200 steps/day) and hyperuricemia.

Mediator	Total effect	*p*-value	Indirect effect (95% CI)	*p*-value	Direct effect (95% CI)	*p*-value	Proportion of mediation	*p*-value
HDL-c	9.28 × 10^247^ (11.1, 7.19 × 10^248^)	**<0.001**	9.31 × 10^246^ (4.43, 7.21 × 10^247^)	**<0.001**	8.35 × 10^247^ (5.33, 6.47 × 10^248^)	**<0.001**	27.6%	**<0.001**
LDL-c	3.10 × 10^242^ (13.2, 2.40 × 10^243^)	**<0.001**	2.48 × 10^239^ (−8.35 × 10^84^, 1.92 × 10^240^)	0.800	3.10 × 10^242^ (13.1, 2.40 × 10^243^)	**<0.001**	2.0%	0.800
TG	1.72 × 10^243^ (14.0, 1.33 × 10^244^)	**<0.001**	1.68 × 10^242^ (3.32, 1.30 × 10^243^)	**<0.001**	1.55 × 10^243^ (8.27, 1.20 × 10^244^)	**<0.001**	21.8%	**<0.001**
TC	1.58 × 10^242^ (14.1, 1.22 × 10^243^)	**<0.001**	−4.96 × 10^238^ (−3.84 × 10^239^, 0.001)	0.600	1.58 × 10^242^ (14.1, 1.22 × 10^243^)	**<0.001**	0%	0.600

The value in bold denotes significant differences (*p* < 0.05). HDL-c, High-density lipoprotein cholesterol; LDL-c, Low-density lipoprotein cholesterol; TC, Total cholesterol; TG, Triglyceride. Analysis was adjusted by age and sex, ethnicity, socioeconomic status, education, employment status, cigarette smoking, vegetable and fruit intake, alcohol consumption, body mass index, sedentary behavior, sleep duration, and total physical activity.

## Discussion

In this study, we observed a significant association between climbing 160–200 steps of stairs daily and a reduced risk of hyperuricemia. The relationship between stair climbing and decreased hyperuricemia risk was particularly pronounced in specific subgroups, including participants aged 45–65 years, male individuals, those with a normal BMI, and those with a history of alcohol consumption. Additionally, this effect was mediated by the levels of HDL-c and triglyceride, while the mediation effects of total cholesterol and LDL-c were not significant.

While various forms of exercise have been studied, our findings emphasize the potential benefits of stair climbing in a range of 160–200 steps per day. Hyperuricemia is closely associated with obesity, insulin resistance, and metabolic syndrome, all of which exacerbate uric acid accumulation in the bloodstream (26–28). Moreover, climbing stairs might enhance basal metabolic rate and insulin sensitivity (29), leading to increased caloric expenditure and a reduction in visceral fat, as well as protect against the metabolic syndrome (14). However, these effects remain hypothetical in our cohort as these parameters were not directly assessed. In addition, no significant association was observed in the high-exposure group (>200 steps/day), which may reflect that excessive stair climbing could induce joint stress in susceptible individuals, potentially counteracting its metabolic benefits—a phenomenon analogous to the uric acid fluctuations documented in marathon runners (30). Our results are consistent with previous studies that have suggested physical activity as a protective factor against hyperuricemia (7, 9–11, 31–33). Although the intensity and duration of exercise vary among studies, our findings demonstrated that climbing 160–200 steps of stairs per day can have a meaningful impact on lowering the risk of hyperuricemia. To translate our findings into practice, multi-level interventions are needed. For instance, placing eye-catching signage near elevators may nudge behavior change. Coupling this with mobile app tracking (e.g., recording stair counts via accelerometers) could reinforce adherence through self-monitoring.

For participants aged 45–65 years, the relationship between stair climbing and reduced hyperuricemia risk may be attributed to the fact that this age group is more likely to experience a decline in muscle mass and an increase in body fat, which can exacerbate the development of hyperuricemia (34). Stair climbing, as a weight-bearing and moderate-intensity exercise, may help mitigate these changes by improving muscle strength (35, 36), enhancing metabolic rate (37), and promoting better lipid metabolism (29, 38), thereby lowering serum uric acid levels. The stronger association found in male participants might be explained by sex differences in uric acid metabolism (39). Men typically have higher serum uric acid levels than women due to differences in renal clearance, hormonal influences, and body composition (40–44). Regular physical activity has been shown to improve renal function and uric acid excretion (45–48), which could be particularly beneficial for men in this age range, who are at a higher risk for hyperuricemia and gout. Among participants with a normal BMI, the beneficial effects of stair climbing on hyperuricemia risk could be related to the fact that these individuals are less likely to have obesity-related metabolic disturbances (49), which can contribute to elevated uric acid levels. Normal BMI individuals may experience more pronounced improvements in metabolic function and uric acid clearance from physical activity such as stair climbing, without the confounding effects of obesity. Alcohol, particularly beer and spirits, is known to increase uric acid production and decrease renal excretion (50). However, physical activity has been shown to improve the clearance of uric acid through the kidneys (51–54) and reduce the inflammatory response (55–57). Therefore, engaging in regular stair climbing may help attenuate the hyperuricemic effects of alcohol consumption by enhancing renal excretion and mitigating inflammation.

The relationship between stair climbing and hyperuricemia reduction is likely multifactorial, and our study offers further insight into the possible mechanisms involved. Although the mediation effects of HDL-c and triglyceride were modest, their involvement underscores the complex interplay between lipid metabolism and uric acid homeostasis. The role of HDL-c in this mediation process is consistent with its known protective effects on cardiovascular health (58–60) and metabolic function (61). HDL-c is believed to facilitate the removal of excess cholesterol from peripheral tissues, a mechanism that may also help in lowering uric acid levels and reducing the risk of hyperuricemia. Similarly, triglyceride levels, which are often elevated in metabolic dysfunction, may contribute to the pathogenesis of hyperuricemia (62–64). The relationship between physical activity and lipid metabolism is well-documented, with regular exercise known to improve lipid profiles (65–67). Our findings align with this body of evidence, suggesting that the beneficial effects of stair climbing on lipid metabolism could, in part, underlie the observed reduction in hyperuricemia risk.

In contrast, the lack of significant mediation by LDL-c and total cholesterol is intriguing. While LDL-c and total cholesterol are traditionally associated with cardiovascular risk (68, 69), our findings suggest that their role in the development of hyperuricemia may not be as direct or pronounced in the context of stair climbing. This finding may indicate that specific lipid fractions, particularly HDL-c and triglycerides, are more directly involved in influencing uric acid metabolism in response to physical activity. Our findings support the hypothesis that daily stair climbing can indirectly influence uric acid levels by improving lipid profiles. This reinforces the importance of incorporating simple, accessible forms of exercise into daily routines as part of a broader strategy for preventing and managing hyperuricemia.

### Strengths and limitations

One of the strengths of our study is the focus on a specific physical activity, stair climbing, which offers a practical and accessible form of exercise for individuals at risk of hyperuricemia. It is particularly notable that only a specific range of stair climbing activity (160–200 steps per day) was associated with a significant reduction in risk. This finding suggests that, while any physical activity may have some benefits, there may be an optimal intensity or duration for effectively mitigating hyperuricemia risk.

However, several limitations must be considered. First, this is an observational study, and as such, it cannot establish a causal relationship. The mediation analysis we performed shows a potential link between lipid metabolism and the reduced risk of hyperuricemia, but it does not provide direct evidence of causality. Future research studies should consider randomized controlled trials to confirm the role of stair climbing and its impact on lipid profiles and uric acid levels. Additionally, while we controlled for several confounding variables, there may still be unmeasured factors influencing both stair climbing and hyperuricemia risk, such as dietary habits or genetic predispositions. Although we adjusted for general dietary patterns (fruit/vegetable intake), the lack of specific purine-rich food data represents a notable limitation. Future studies should incorporate detailed dietary assessments, such as food frequency questionnaires, specifically targeting purine sources. The generalizability of our findings may also be limited by the specific population studied. While the UK Biobank provides robust phenotypic and genetic data, its underrepresentation of non-white and younger populations may limit the generalizability of our results. Further research is needed to confirm these results in diverse demographic groups.

## Conclusion

In conclusion, our study highlights the potential for daily stair climbing (160–200 steps/day) as an effective strategy for reducing hyperuricemia risk. This association appears partially mediated by improved HDL-c and triglyceride levels, providing valuable insights into the physiological mechanisms underpinning this association. Future experimental studies should validate these findings and clarify the underlying mechanisms.

## Data Availability

The datasets presented in this study can be found in online repositories. The names of the repository/repositories and accession number(s) can be found at: Detailed information about the UK Biobank cohort is available on their website (www.ukbiobank.ac.uk). All participants have signed informed consent forms, and the application number of UKB in our study was 75,732.

## Footnotes

^1^ www.ukbiobank.ac.uk
